# Discoloration of Gold Fillings

**Published:** 1859-07

**Authors:** J. D. Wingate


### DISCOLORATION OF GOLD FILLINGS.

THE patient occasionally asks,—If dentists fill teeth with pure gold as they say they do—why do some of the plugs turn black?

Never having heard any cause assigned, I shall give what I think is the leading one. It is the abrasion of the steel instruments used in inserting the filling into the tooth; small particles of the steel incorporating themselves with the gold, and by oxydation in the mouth, form a coating over the plug, which by a little polishing is easily removed, leaving the filling as bright as ever; but the patient has a right to know why the discoloration. We find inventions and inventors daily increasing. Could none of these find out a metal, from which to make pluggers that would have all the required properties for filling teeth, without spoiling the beauty of the filling? J. D. WINGATE.

